# Molecular characterization of *VP1* gene during the foot and mouth disease virus outbreak in East Java, Indonesia, in 2022

**DOI:** 10.14202/vetworld.2024.2469-2476

**Published:** 2024-11-07

**Authors:** Zayyin Dinana, Suwarno Suwarno, Imam Mustofa, Jola Rahmahani, Kusnoto Kusnoto, Aussie Tahta Maharani, Anisa Lailatul Fitria, Adiana Mutamsari Witaningrum, Firdausy Kurnia Maulana, Nur Saidah Said, Deka Uli Fahrodi, Fedik Abdul Rantam

**Affiliations:** 1 Doctoral Program of Veterinary Science, Faculty of Veterinary Medicine, Universitas Airlangga, Surabaya, Indonesia; 2 Division of Microbiology, Faculty of Veterinary Medicine, Universitas Airlangga, Surabaya, Indonesia; 3 Division of Reproduction, Faculty of Veterinary Medicine, Universitas Airlangga, Surabaya, Indonesia; 4 Division of Parasitology, Faculty of Veterinary Medicine, Universitas Airlangga, Surabaya, Indonesia; 5 Department of Viral Diarrhea, Institute of Tropical Disease, Universitas Airlangga, Surabaya, Indonesia; 6 Division of Veterinary Public Health, Faculty of Veterinary Medicine, Universitas Airlangga, Surabaya, Indonesia; 7 Airlangga Disease Prevention and Research Center, Universitas Airlangga, Surabaya, Indonesia; 8 Department of Animal Husbandry, Faculty of Animal Husbandry and Fisheries, Universitas Sulawesi Barat, Majene, Indonesia; 9 Research Center for Vaccine Technology and Development, Institute of Tropical Disease, Universitas Airlangga, Surabaya, Indonesia

**Keywords:** cattle, foot and mouth disease virus, Indonesia, phylogenetic tree, viral proteins 1

## Abstract

**Background and Aim:**

Foot and mouth disease (FMD) is highly contagious in cloven-hoofed animals, and it causes outbreaks in Indonesia and several countries worldwide. This disease is caused by the FMD virus (FMDV), which belongs to the genus Aphthovirus and family Picornaviridae. In 1990, the World Organization for Animal Health Office International des Epizooties recognized Indonesia as an FMD-free country. A new FMDV outbreak in Indonesia was reported in April 2022 and confirmed in May 2022, resulting in economic losses to the beef cattle sector. This study aimed to determine the genotype and amino acid content of viral proteins (*VP1*) gene.

**Materials and Methods:**

Samples were obtained from vesicle swabs from the mouth and feet of cattle in Banyuwangi Regency, Lamongan Regency, and Surabaya City, East Java, Indonesia. Samples were identified using one-step reverse transcriptase-polymerase chain reaction with a pair of specific primers encoding the *VP1*O serotype with a target of 1165 bp.

**Results:**

Sequencing revealed that the FMDV subtype belonged to the O/ME-SA/Ind2001e. Phylogenetic analysis showed that our isolate was 100% amino acid-identical to the Indonesian outbreak isolates from 2022 and 95% identical to isolates from Southeast Asia. The amino acid substitutions found in the G-H Loop of the *VP1*were S134C, D138E, T140A, and A156T. Only the K135Q mutation was detected in Lamongan.

**Conclusion:**

The spread of the subtype O/ME-SA/Ind2001e in South-east Asia caused an outbreak in Indonesia due to less stringent animal traffic control measures. Surveillance studies and whole-genome sequence analyses are important for monitoring FMDV genetics in Indonesia.

## Introduction

Foot and mouth disease (FMD) is a highly contagious animal disease in cloven-hoofed ruminants, with a rapid transmission rate worldwide [1]. According to the World Organization for Animal Health (WOAH), FMD is included in the list of infectious animal diseases with 100% morbidity in FMD-confirmed animal populations and a mortality rate of 20%–30% in young animals and 1%–5% in adult animals [2]. FMD outbreaks have been widely reported, and FMD virus (FMDV) endemic areas are found in Africa and Asia. FMDV belongs to the genus Aphthovirus and family Picornaviridae and is a single-stranded RNA with a positive genome of 8500 nucleotides and a very low molecular weight in the range of 7.2–8.4 kb with a diameter of 25–30 nm [3, 4]. The FMDV is surrounded by structural viral proteins (VP1, VP2, VP3, and VP4) and non-structural (Lpro, 2A, 2B, 3A, 3B, 3Cpro, and 3Dpro) proteins [3]. FMDV is characterized by high genetic variability, and there are seven different serotypes worldwide: O, A, C, Asia 1, Southern African Territories (SAT) 1, SAT 2, and SAT 3 [4]. The VP1-coding nucleotide sequence has been used to genetically characterize FMDV strains [5].

In 1889, FMDV outbreaks spread to various livestock sectors in Indonesia, and in 1990, WOAH recognized Indonesia as an FMD-free country without vaccination [6]. However, in April 2022, an FMDV outbreak was detected in Indonesian cattle and goat farming. The first case of FMD in Indonesia was confirmed on April 28, 2022. This was confirmed in the Decree of the Minister of Agriculture of the Republic of Indonesia, which regarding the identification of FMD outbreak areas in the provinces of Aceh and East Java [7]. The absence of cross-reactivity protection against FMD viruses makes it better to use vaccines in areas where outbreaks occur [6]. FMDV outbreak cases in Indonesia were serotypes O/ME-SA/Ind-2001e [8]. Based on the observed outbreaks, further research is needed to determine the nucleotide and amino acid sequences of FMD viruses circulating in Indonesia. Detection and confirmation of FMD distribution are essential in establishing diagnosis; therefore, an accurate and rapid reverse transcriptase-polymerase chain reaction (RT-PCR) method and confirmation of the virus strain through sequencing are needed.

This study aimed to determine the genotype of FMDV in East Java, Indonesia and relationship based on phylogenetic tree analysis. Also to find the nucleotide and amino acid changes in East Java isolates compared to vaccine strains used in Indonesia.

## Materials and Methods

### Ethical approval

This study was approved by the Ethics Committee of the Faculty of Veterinary Medicine, Airlangga University, Surabaya, Indonesia (approval number: 1.KEH.096.07.2024).

### Study period and location

This study was conducted from September to December 2022. Samples were collected from vesicle swabs of lesions in the mouth and feet of cattle from smallholder farms in three areas of East Java, Indonesia (Lamongan, Surabaya, and Banyuwangi).

### Sample collection

We collected 25 vesicle swab samples from cattle with typical clinical symptoms of early FMDV infection (elevated temperature, salivation, depression, sores, and blisters on the mouth and feet). Swab samples were collected and placed into 5-mL sterile tubes containing viral transport media (phosphate buffer saline, pH 7.4 and 1% antibiotic). All collected samples were shipped in a cool ice pack (4°C). Samples were stored in a –80°C freezer in the laboratory until analysis.

### RNA extraction and one-step RT-PCR

Viral RNA was extracted from 140 μL suspension using a QIAmp Viral RNA Mini Kit according to the manufacturer’s instructions (Qiagen, Hilden, Germany). RNA was eluted with 60 μL of nuclease-free water and stored at –80°C before use. RNA was isolated using a one-step RT-PCR (AMV) kit (Takara Bio Inc, Japan). Detection of FMDV serotype O in the *VP1* gene using one pair of primers, forward primer O1C244F (5’-GCAGCAAAACACATGTCAACACCTT-3’) and reverse primers EUR2B52R (5’-GACATGTCCTC CTGCATCTGGTTGAT-3’) was performed according to a published protocol [9, 10]. A total of 15 μL of RT-PCR volume consisting of 2 μL of RNA, 5 μL of reaction mix, 0.75 μL of enzyme mix, and 4.25 μL of RNase-free water were mixed with 1.5 μL of each primer (5 μM) was used. One-step RT-PCR cycles were performed using the following conditions: 30 min at 42°C (reverse transcription step), 5 min at 94°C followed by 35 cycles at 94°C for 60 s, 55.5°C for 60 s, 72°C for 60 s, and 72°C for 10 min. The PCR products were stored in a refrigerator before electrophoresis. RT-PCR products were analyzed using 2% agarose gel electrophoresis with 1× Tris Borate ethylenediaminetetraacetic acid buffer and stained with ethidium bromide. RT-PCR products (5 μL) were collected and mixed with 1 μL DNA staining, and a DNA 2 μL ladder was placed into the wells. DNA fragments from the positive samples were visualized using the Gel Doc device (Axygen®, Corning, USA) under ultraviolet light.

### Nucleotide sequencing and phylogenetic analysis

Sequences were determined directly from positive RT-PCR products using the BigDye Terminator v3.1 cycle sequencing kit and an Applied Biosystems 3500xL Genetic Analyzer (Applied Biosystems, Waltham, MA, USA). Phylogenetic analyses were performed using amplified VP1 nucleotide sequences. The reference sequences were retrieved from the DNA Data Bank of the GenBank database (www.ncbi.nlm.nih.gov). Alignments were performed using ClustalW software, and phylogenetic tree analysis was performed using the maximum composite likelihood method with the neighbor-joining method. The analysis was validated with 1000 bootstrap replicates with bootstrap values >70% displayed on the phylogenetic tree. These analyses were conducted using the Molecular Evolutionary Genetic Analysis (MEGA) version X software [11].

### GenBank accession number

The gene sequences described in this study have been deposited in GenBank under accession numbers PP066264 and PP066270.

## Results

**Figure-1:**
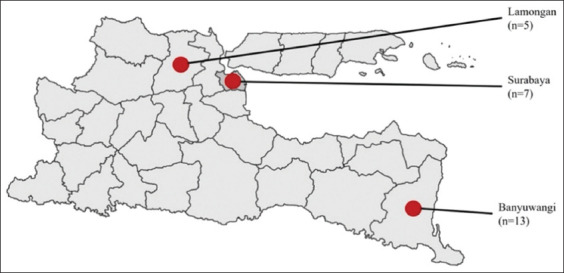
Geographical location of sample areas in East Java, Indonesia (red circles) and distribution of sampling size in Lamongan (5), Surabaya (7), and Banyuwangi (13). [Source: https://id.m.wikipedia.org/wiki/Berkas: Peta_jawa_timur.png].

**Figure-2:**
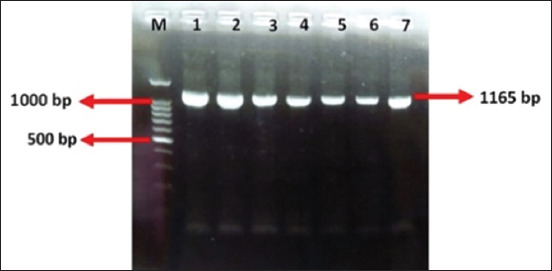
Amplification of the *VP1* gene fragment (1165 bp) using one-step reverse transcriptase-polymerase chain reaction of the FMDV virus product using O serotype VP1 gene primers; M DNA Ladder (100bp); lanes 1–7 PCR products. Line 1–3 samples from Banyuwangi, line 4 and 5 from Lamongan; lines 6 and 7 from Surabaya. FMDV=Foot and mouth disease virus, VP1=Viral proteins 1, PCR=Polymerase chain reaction.

Figure-1 shows the three locations in East Java, Indonesia, used for FMDV sampling. The amplification results of the RT-PCR products using serotype O-specific primers for *VP1* revealed DNA bands in seven samples (Figure-2). The *VP1* gene fragment was obtained by visualizing 1165-bp DNA bands with a length of 1165 bp (Figure-2). Serotype O was confirmed by Sanger sequencing of the RT-PCR products.

**Figure-3:**
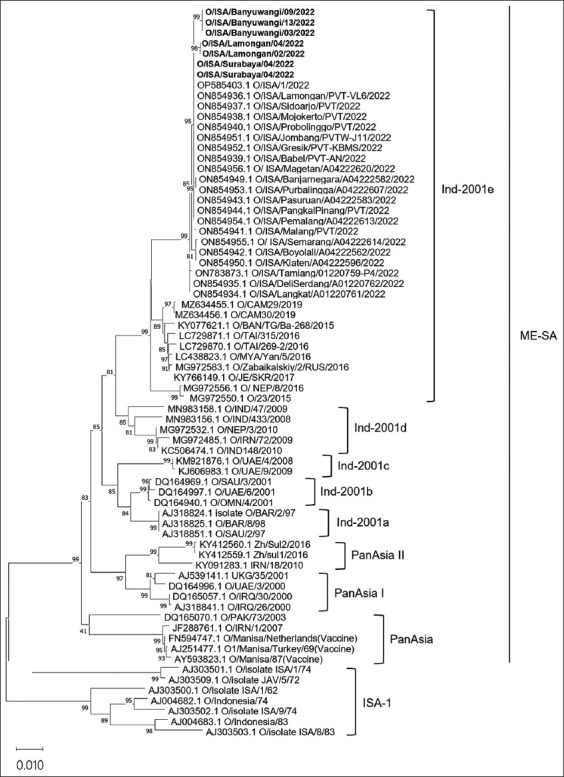
Maximum clade credibility tree showing the phylogeny of the FMDV serotype O ME-SA Ind-2001e. Phylogenetic tree analysis of FMDV based on the *VP1* gene. The FMDV strains identified in this study are shown in bold font. The phylogenetic tree was constructed using the Neighbor-joining Maximum Composite Likelihood method and validated using 1000 bootstrap replicates. Bootstrap values ≥70% are shown for the branches. FMDV=Foot and mouth disease virus, VP1=Viral proteins 1.

Phylogenetic tree analysis was performed based on the nucleotide sequence of FMDV *VP1*. Seven representative FMDV belonged to the topotype of the ME-SA lineage Ind-2001e and clustered with previous samples in Indonesia (Figure-3). Homology analysis showed that ISA/Surabaya/02/2022 and ISA/Surabaya/04/2022 shared 100% nucleotide and amino acid identity with strains OP585403, ON854936, ON854937, ON854938, ON854939, ON854940, ON854941, ON854943, ON854944, ON854949, ON854951, ON854952, ON854953, ON854954, and ON854956 (Table-1). Isolates ISA/Lamongan/02/2022, ISA/Lamongan/03/2022, ISA/Banyuwangi/03/2022, ISA/Banyuwangi/09/2022, and ISA/Banyuwangi/13/2022 showed 99.8% nearest nucleotide and amino acid identities with strain OP585403 (Table-1).

**Table-1:** Sequence identities between the FMDV strains in this study and reference strains were obtained through sequence comparisons.

Accession number	Strain	Lineage	Nucleotide identity (%)			Amino acid identity (%)		
	
			Banyuwangi	Lamongan	Surabaya	Banyuwangi	Lamongan	Surabaya
AY593823	O/Manisa/87	PanAsia	92.5	92.8	93.5	94.2	94.8	95.2
FN594747	O/Manisa/Netherlands	PanAsia	92.8	93.2	93.4	94.7	95.1	95.7
AJ251477	O1/Manisa/Turkey/69	PanAsia	92.6	93.2	93.6	94.5	95.2	95.6
OP585403	O/ISA/2022	Ind-2001e	99.8	99.8	100	99.8	99.8	100
ON854936	O/Lamongan/2022	Ind-2001e	98.2	99.1	100	99.8	99.8	100
ON854937	O/Sidoarjo/2022	Ind-2001e	97.2	99.1	100	99.7	99.8	100
ON854935	O/DeliSerdang/2022	Ind-2001e	97.2	98.2	99.1	99.7	99.7	99.8
ON783873	O/Tamiang/2022	Ind-2001e	97.2	98.2	99.1	99.8	99.7	99.8
ON854938	O/Mojokerto/2022	Ind-2001e	98.2	99.1	100	99.8	99.8	100
ON854939	O/Babel/2022	Ind-2001e	98.2	99.1	100	99.8	99.8	100
ON854949	O/Banjarnegara/2022	Ind-2001e	98.2	99.1	99.9	99.7	99.8	100
ON854952	O/Gresik/2022	Ind-2001e	98.2	99.1	100	99.8	99.8	100
MZ634455	O/CAM30/2019	Ind-2001e	93.9	94.2	94.9	94.8	94.9	95.2
MN983158	O/IND/47/2009	Ind-2001d	91.5	91.8	92.3	93.4	93.5	93.7
KM921876	O/IND148/2010	Ind-2001c	92.9	93.2	93.7	93.2	93.5	93.7
DQ164969	O/SAU/3/2001	Ind-2001b	92.3	92.6	93.1	93	92.9	93.1
AJ318824	O/BAR/2/97	Ind-2001a	91.9	92.1	92.5	93.3	93.5	93.7
AJ539141	O/UKG/35/2001	PanAsia-II	92.1	92.3	92.8	94.5	94.5	94.7
DQ165057	O/IRQ/30/2000	PanAsia-I	92.3	92.5	93.1	94.4	94.5	94.7
DQ165070	O/PAK/73/2003	PanAsia	91.9	92.2	92.7	92.3	92.5	92.7
AJ004683	O/Indonesia/83	ISA-1	85.8	86.2	86.7	88.2	88.3	88.5

FMDV=Foot and mouth disease virus

A comparative analysis of the nucleotide identity of Indonesia’s previous strain from 1962 to 1983, adrift, clustered with our strain. Nucleotide and amino acid identities with the previous Indonesian strain were 86.7% and 88.5% (Table-1). Our samples clustered with South-east Asian strains, with nucleotide sequence similarities between 95.3% and 95.5%, respectively. Interestingly, the highest nucleotide (94.9%) and amino acid (95.5%) identities were observed in strains from Cambodia (Table-1). Among the reference vaccine strains, the highest nucleotide and amino acid sequence similarity was obtained with the O/Manisa/Netherlands strain (92.8% and 95.7%, respectively), followed by O1/Manisa/Turkey/69 (92.6% and 95.7%, respectively), and O/Manisa/87 (92.5% and 95.2%, respectively) (Table-1).

**Figure-4:**
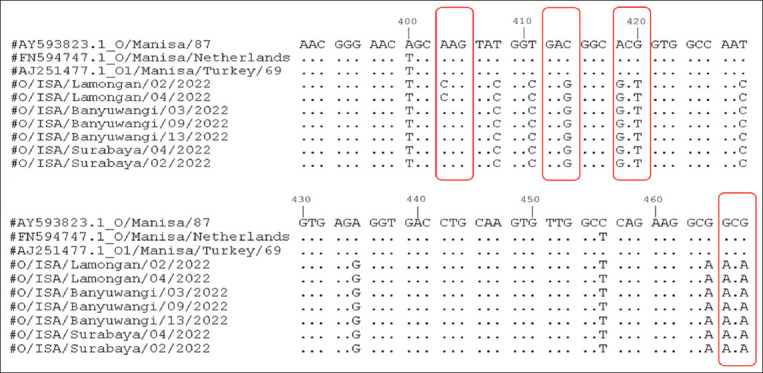
Nucleotide alignment of VP1 gene isolates in this study compared with other FMDV isolates. The red squares denote the major variable sites selected for the comparison.

**Figure-5:**
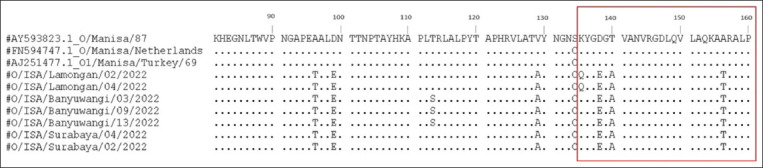
Deduced amino acid sequence alignment of VP1 among the FMDV isolates in this study compared with other FMDV isolates. The red squares denote the major variable sites selected for the comparison. FMDV=Foot and mouth disease virus, VP1=Viral proteins 1.

Genetic analysis was performed by aligning multiple amino acid sequences of *VP1* associated with the immunogenic region. The critical amino acid substitutions at *VP1* G-H loop positions 134–160 are responsible for antigenic heterogeneity. Nucleotide mutations were observed at positions C415G, A418G, G420T, G466A, and G468A (Figure-4). The A403C mutation was detected only in Lamongan samples (Figure-4). Based on the nucleotide mutation, four amino acid substitutions were found in the antigenic variation sites at S134C, D138E, T140A, and A156T (Figure-5) and one substitution was observed in the Lamongan samples at K135Q, respectively (Figure-5). Therefore, the amino acid differences in the antigenic sites between our strain and the vaccine strain may contribute to broad antigenic coverage.

FMDV=Foot and mouth disease virus, VP1=Viral proteins 1.

## Discussion

FMD is an infectious disease that has affected cattle farming in Indonesia since the April 2022 outbreak. A recent article noted that serotype O topotype ME-SA strain Ind-2001e caused an FMDV outbreak in Indonesia [8]. This study reports the molecular characterization of the *VP1* gene sequences of local strains of FMDV isolated from cattle in East Java, Indonesia. One-step RT-PCR was performed using serotype O-specific primers to amplify fragments of the VP1 gene-encoding region of FMDV.

The O/ME-SA/Ind-2001 lineage was first reported in India in 2001 and has since spread to several regions of South-east Asia, the Middle East, and Africa [12]. Since their emergence worldwide, they have been classified into five sublineages (a–e) [13]. In South-east Asia, O/ME-SA/Ind-2001e was reported in Cambodia [14], Myanmar [15], China [16], and Thailand [17]. FMD cases are endemic to beef cattle farms in Cambodia, and in 2019, serotype O/ME-SA/Ind2001e was found in 15.8% of cases [14]. The spread of FMD in South-east Asia has caused the outbreak in Indonesia due to less strict animal traffic control.

The FMDV vaccine was introduced in Indonesia in June 2022 and distributed in several districts [3]. A total of 11,970,572 beef cattle, dairy cattle, buffaloes, goats, and sheep were vaccinated from June to December 2022 [18]. Nucleotide identity was compared between vaccine strains circulating in Indonesia (>95%). Vaccine compatibility tests against Ind2001d strain isolates showed that the O/ME-SA/PanAsia vaccine was compatible with the O/ME-SA/Ind2001d strain isolates [19]. However, no vaccine compatibility tests have been conducted between the O/ME-SA/PanAsia vaccine strain used in Indonesia and the 2022 Indonesian isolate.

Phylogenetically, O/ME-SA/Ind2001e is closely related to the isolates from the Indonesian outbreak that occurred in April 2022. Surabaya isolates have 100% amino acid identity with Indonesian isolates that were previously circulated in 2022. However, the Banyuwangi and Lamongan isolates have more than 99% amino acid identity similarity and are clustered with Indonesian isolates from 2022. The isolates in this study had 88.5% amino acid identity with Indonesian isolates from 1980s and exhibited topotype differences. The O/ME-SA/Ind-2001 e strain was first detected in several regions of Indonesia based on the expression of *VP1* gene. The origin of the O/ME-SA/Ind-2001e genotype circulating in Indonesia could not be determined based on a comparison of the *VP1*-encoding gene with strains circulating in surrounding countries, but the spread of FMDV can be assumed to be due to introductions from the nearest countries that have the same epidemiological history as the emergence of the FMDV strain O/ME-SA/Ind-2001e.

Several amino acid mutations were found in the G-H Loop of the *VP1* encoding gene compared with the vaccine strain. Nucleotide changes caused by the viral adaptation system in host cells are influenced by microenvironmental factors (epigenetics), including changes in gene expression [20]. A single nucleotide mutation at position C415G resulting in an amino acid substitution change of S134C, serine→cysteine has been reported to disrupt the 3C protease system (3C Pr°) involved in polyprotein processing in picornaviruses [21]. Nucleotide mutation at position A418G changes the amino acid in region D138E (aspartic acid→glutamine acid) and affects viral interaction with alternative cellular receptors and viral infection in cells [22, 23]. The amino acid substitution D138G in *VP1* has also been reported to be crucial in the response to integrin-independent infection by a few genetic and engineered FMDVs (Cathay topotype, serotype O) in baby hamster kidney-21 (BHK-21) and Chinese hamster ovary (CHO) cell lines [24]. G420T nucleotide mutation changes the amino acid residue T140A (thorine→alanine), affects the maintenance of FMDV polymerase fidelity, and ensures faithful replication of the FMDV genome [25, 26]. The two nucleotide mutations G466A and G468A cause amino acid substitution at position A156T (alanine→thorine) and are responsible for the adaptation and interaction of the virus with cellular receptors due to changes in the surface of the viral capsid [27]. Changes in the G-H loop region and C-terminal residues have been reported to be associated with changes in viral antigenicity [28, 29]. Several amino acid substitutions were also found in the Egyptian isolates compared with the vaccine strains S134A, S137T, T139G, G141S, and A156T [27]. Substitutions in the G-H Loop region can cause incompatibility with the current vaccine strains used for vaccination [30]. However, no reports detail the efficacy of the currently available vaccines in Indonesia.

These findings indicate the importance of surveillance studies and molecular analyses in monitoring FMD cases in Indonesia. In summary, the genetic characteristics of FMDV serotype O circulating in Indonesia exhibit nucleotide variation in the antigenic region (G-H Loop). This result indicates that the virus isolated from East Java is the same strain as the previous outbreak strain but differs from the circulating vaccine strain. Because this study only analyzed the *VP1* fragment-encoding gene, whole-genome molecular FMDV analysis of Indonesia isolates is necessary.

## Conclusion

Phylogenetic analysis revealed that the FMDV isolate from East Java was highly homologous to Indonesian. Amino acid variations were observed at positions S134C, D138E, T140A, and A156T. The discovery of amino acid mutations in *VP1* indicates that genomic analysis of other (*VP2, VP3*, and *VP4*) is important for determining vaccine effectiveness. Therefore, further research is needed regarding whole-genome molecular analyses based on amino acid differences between FMDV isolates circulating in Indonesia.

## Authors’ Contributions

ZD, AMW, FKM, NSS, and DUF: Collected samples. ZD, ATM, ALF, and FKM: Performed the experiments and data analysis. ZD: Drafted the manuscript. FAR, JR, and SS: Supervised the study. FAR, SS, IM, JR, and KK: Drafted the manuscript. All authors have read and approved the final manuscript.
